# Wu’s seven steps for endoscopic central and lateral neck dissection via breast combined with oral approach for papillary thyroid cancer

**DOI:** 10.1007/s00464-023-09910-1

**Published:** 2023-04-03

**Authors:** Yuanyuan Wang, Yezhe Luo, Guoyang Wu, Suqiong Lin, Yilong Fu

**Affiliations:** 1 Department of Thyroid Surgery, Zhengzhou University First Affiliated Hospital, Zhengzhou, 450000 China; 2 Department of General Surgery, ZhongShan Hospital of Xiamen University, Xiamen, 361004 China

**Keywords:** Papillary thyroid cancer, Endoscopic thyroidectomy, Neck dissection

## Abstract

**Objective:**

We had previously reported endoscopic central and lateral neck dissection via breast combined with an oral approach for papillary thyroid cancer treatment. In this study, we optimized the procedure with Wu’s seven steps to make the procedure quicker and easier.

**Methods:**

Wu’s seven steps for endoscopic central and lateral neck dissection via breast combined with oral approach for papillary thyroid cancer are: (1) establish the working space, (2) isolate the sternocleidomastoid and internal jugular vein, (3) dissect the thyroid via breast approach, (4) dissect the central lymph nodes via oral approach, (5) dissect the inferior board of level IV via oral approach, (6) remove the tissues of levels IV, III, and II via breast approach, and (7) wash the working space and place drainage tubes. Twelve patients were assigned to the Wu’s seven steps group, and 13 patients were assigned to the contrast group. The operative procedure of the contrast group was the same as Wu’s seven steps except for a few key differences, such as that the central lymph nodes were dissected via breast approach first and the internal jugular vein(IJV) was dissected from the cricoid cartilage down to the venous angle.

**Results:**

The Wu’s seven steps group had a short operation time and few injuries of the internal jugular vein. There were no statistical differences in other clinicopathological features or surgical complications.

**Conclusion:**

It appears that Wu’s seven steps for endoscopic central and lateral neck dissection via breast combined with oral approach for papillary thyroid cancer are effective and safe.

Papillary thyroid cancer is becoming increasingly common in young and middle-aged women [1]. Central and lateral neck dissection could reduce the risk of local recurrence in patients with suspected lymph node metastasis [2]. Endoscopic thyroidectomy and lymph node dissection have developed rapidly. For the dissection of the central compartment, multiple approaches can be used, including the breast approach, trans-oral approach [3], and axillary approach [4]. For endoscopic lateral neck dissection, the breast approach is by far the most common choice [5, 6]. Because lymph nodes located in level VII and the inferior area of level IV are blocked by the sternal manubrium and clavicle, the question of whether these lymph nodes can be dissected completely remains. In a previous attempt to solve this problem, we tried the endoscopic central and lateral neck dissection via breast combined with oral approach for papillary thyroid cancer, but the operation time was too long [7]. Therefore, in the present study, our objective was to find an ideal procedure to make this operation easier and safer. Based on several cases, we optimized the steps of endoscopic central and lateral neck dissection via breast combined with oral approach for papillary thyroid cancer, and we named these steps Wu’s seven steps. We compared the clinical data and surgical outcomes between 12 cases of patients treated with Wu’s seven steps and 13 cases of patients treated with a contrast procedure.

## Materials and methods

### Selection of patients

Between May 2018 and April 2021, 25 papillary thyroid cancer patients with lateral lymph node metastasis underwent endoscopic central and lateral neck dissection via breast combined with oral approach. 12 patients were treated with Wu’s seven steps, and the other 13 patients were treated with a contrast procedure. All the patients were operated on by the same surgeon (Prof. Guoyang Wu) at the General Surgery Department of Zhongshan Hospital at Xiamen University. Patients were investigated by ultrasound and enhanced computed tomography (CT). The diagnosis of papillary thyroid cancer was confirmed by means of preoperative ultrasound-guided fine needle aspiration cytology. Laryngoscopy was performed before each surgery to assess the status of recurrent laryngeal nerves. Only patients who underwent dissection of ipsilateral levels II, III, and IV, total thyroidectomy, and central compartment dissection were selected. The study protocol was approved by Zhongshan Hospital of XiamenUniversity ethics and scientific review board (2021-078). Written informed consent weren obtained from all the participants. We confirm that all methods were performed in accordance with the relevant guidelines and regulations.

### Data availability

The datasets used and/or analysed during the current study available from the corresponding author on reasonable request.

### Inclusion and exclusion criteria

The inclusion criteria were as follows: (1) papillary thyroid cancer with confirmed lymph node metastasis, (2) a highly cosmetic demand, (3) thyroid tumor size of < 2 cm without severe invasion; and (4) largest metastatic lymph node diameter of < 2.0 cm. All methods were carried out in accordance with relevant guidelines and regulations.

The exclusion criteria were as follows: (1) previous neck surgical history, (2) metastatic lymph nodes in the level I or V region, (3) metastatic lymph nodes fused or fixed in the neck, (4) invasion of surrounding tissues, (5) distant metastases, (6) no cosmetic demand, (7) inability to tolerate anesthesia or operation, (8) oral abscess, (9) previous radiation to the head or neck, (10) tracheal or esophageal invasion, (11) recurrent laryngeal nerve palsy, (12) uncontrolled hyperthyroidism [6].

### Operative procedure

Wu’s seven steps for endoscopic central and lateral neck dissection via breast combined with oral approach for papillary thyroid cancer treatment were performed on the 12 patients in the Wu’s seven steps group, described as follows:

1. Working space was established.

**Fig. 1:**
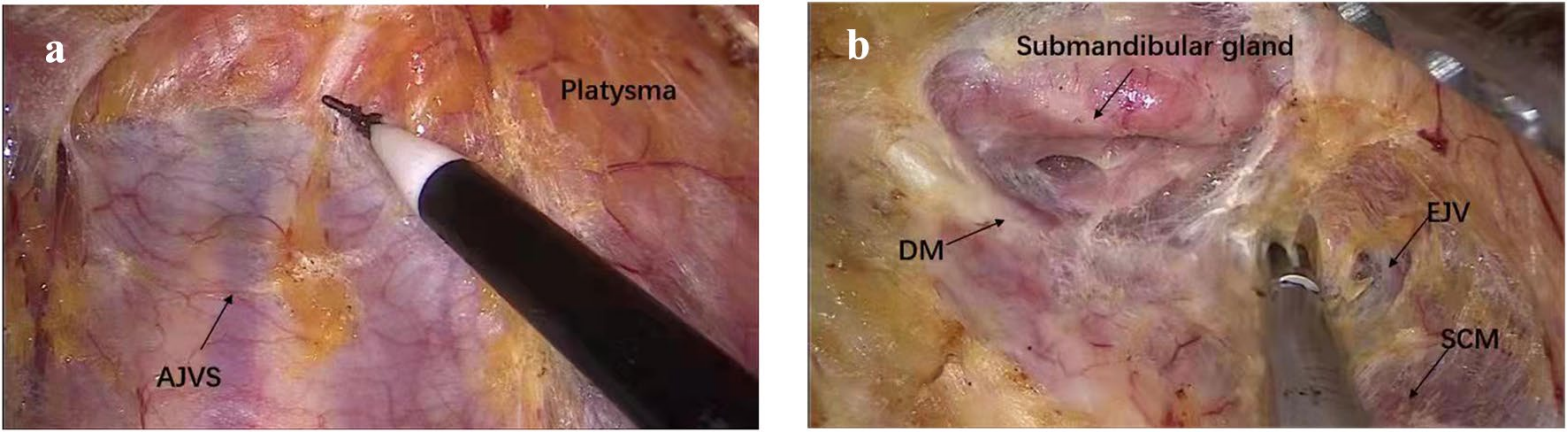
Step 1: working space is established. **a** Working space is established between platysma and AJVS. **b** The submandibular salivary gland and digastric muscle as the superior edge of lateral neck compartment. *AJVS* anterior jugular venous system, *SCM* sternocleidomastoid muscle, *EJV* external jugular vein, *DM* digastric muscle

The patient was placed in the supine position with the neck slightly extended. A 12 mm incision was made parasternally at the nipple level. Through the incision, one 10 mm trocar was inserted into the subcutaneous layer of the anterior chest [8, 9], and three 5 mm trocars were inserted through the left and right areola and left axillary, respectively (Fig. 1). CO_2_ gas was insufflated at 8 mmHg. A 10 mm 30° laparoscope was inserted through the 10 mm trocar. The subcutaneous loose connective tissue was separated, the initial working space was established, and the working space range was dissected to the lateral edge of the posterior border of the sternocleidomastoid (SCM) and to the superior edge of the submandibular salivary gland and digastric muscle.

2 The SCM was isolated, and the internal jugular vein (IJV) and accessory nerve were exposed.

**Fig. 2:**
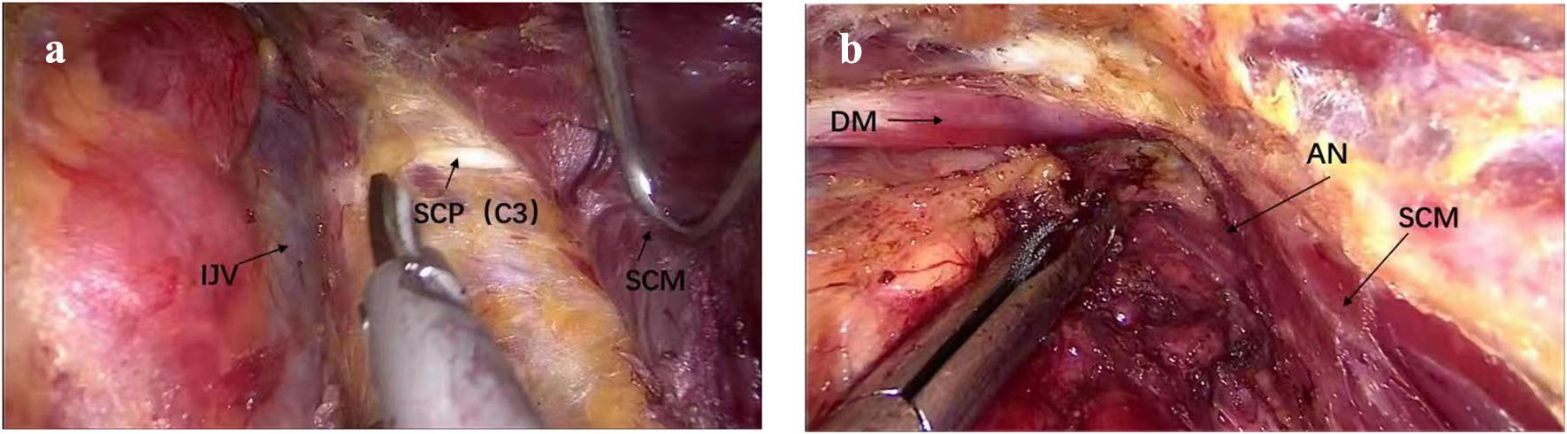
Step 2: the sternocleidomastoid and internal jugular vein are isolated. **a** The internal jugular vein (IJV) was exposed. **b** The accessory nerve was exposed. *SCP* superficial cervical plexus. *IJV* internal jugular vein, *SCM* sternocleidomastoid muscle, *DM* digastric muscle, *AN* accessory nerve

The patient’s head was turned to the opposite side from the lesion to expose the lateral neck compartment area (Fig. 2). The lateral neck compartment was dissected to the lateral edge of the posterior board of the SCM, and the accessory nerve and cervical plexus were exposed. Then, the omohyoid muscle was isolated. To expose the anterior and external boundaries, the IJV was carefully dissected from the venous angle up to the cricoid cartilage along the external border of the sternohyoid muscle. The posterior of level IV was also partly dissected to expose the transverse cervical artery for the convenience of operation in step 5.

3 The thyroid was dissected via breast approach.

**Fig. 3:**
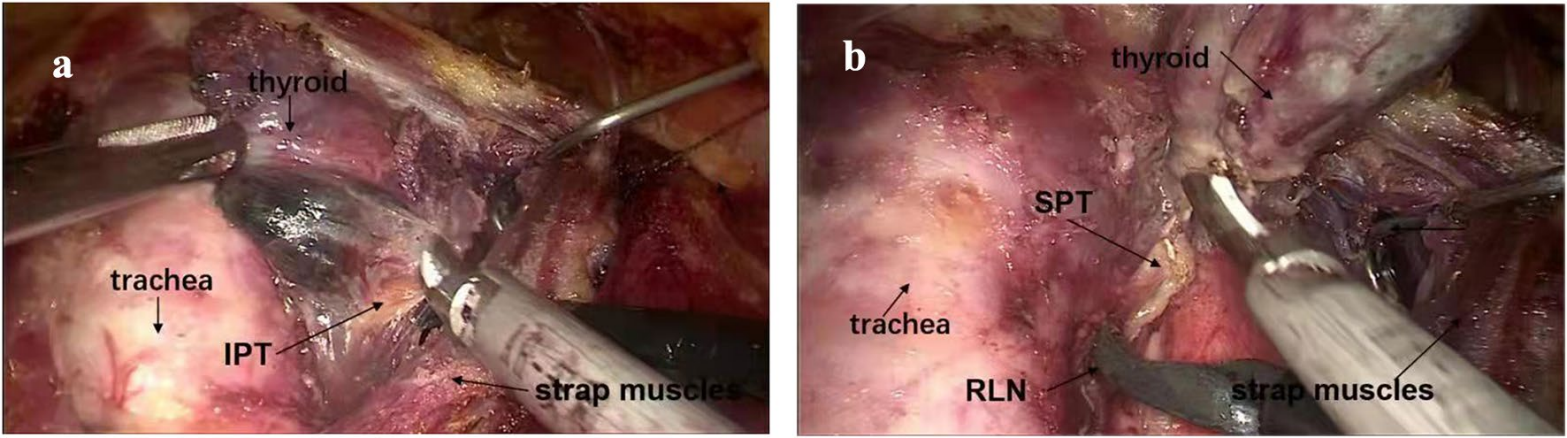
Step 3: the thyroid was dissected via breast approach. **a** Thyroid is dissected via breast approach. **b** The recurrent laryngeal nerve (RLN) and superior parathyroid gland (SPT). *IPT* inferior parathyroid gland, *SPT* superior parathyroid gland, *RLN* recurrent laryngeal nerve

The patient’s head was returned to the neutral position. This procedure was similar to endoscopic thyroidectomy with breast approach (Fig. 3).

4 Central lymph nodes were dissected via oral approach.

**Fig. 4:**
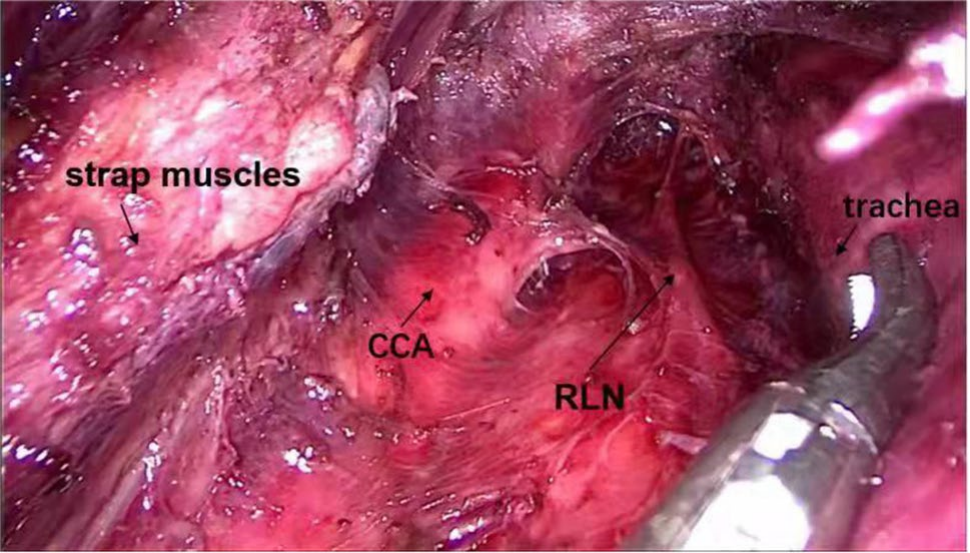
Step 4: central lymph nodes were dissected via oral approach. *CCA* common carotid artery, *RLN* recurrent laryngeal nerve

One 10 mm trocar in the middle and two 5 mm trocars on both sides were inserted into the working space according to the vestibule approach (Fig. 4). Because the working space had been established, the three trocars could be inserted easily with shorter incisions and less injury compared with the trans-oral vestibule approach. The procedure of central compartment dissection was similar to the trans-oral vestibule approach.

5 The inferior border of level IV was dissected via oral approach.

**Fig. 5:**
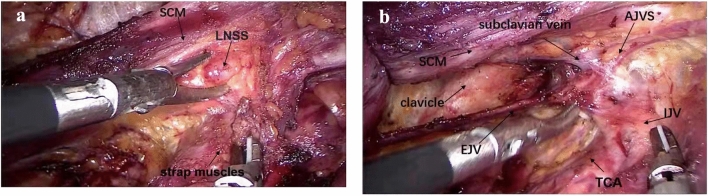
Step 5: the inferior board of level IV is dissected via oral approach. **a** The lymph node between sternocleidomastoid and sternohyoid muscle (LNSS). **b** The venous angle was carefully dissected along the surface of the subclavian vein via oral approach. *SCM* sternocleidomastoid muscle, *LNSS* lymph node between sternocleidomastoid and sternohyoid muscle, *AJVS* anterior jugular venous system, *EJV* external jugular vein, *TCA* transverse carotid artery, *IJV* internal jugular vein

The patient’s head was turned to the opposite side from the lesion again (Fig. 5). From the trocar in the vestibule, the venous angle was carefully dissected along the surface of the subclavian vein to the external border of the SCM. The branch of the thoracic duct on the left side or the lymphatic trunk on the right side was clamped with a 5 mm hemo-lock. Some tissues of level V were dissected by pulling from level IV.

6 The tissues of levels IV, III, and II were removed via breast approach.                                                                                                                                                             

**Fig. 6:**
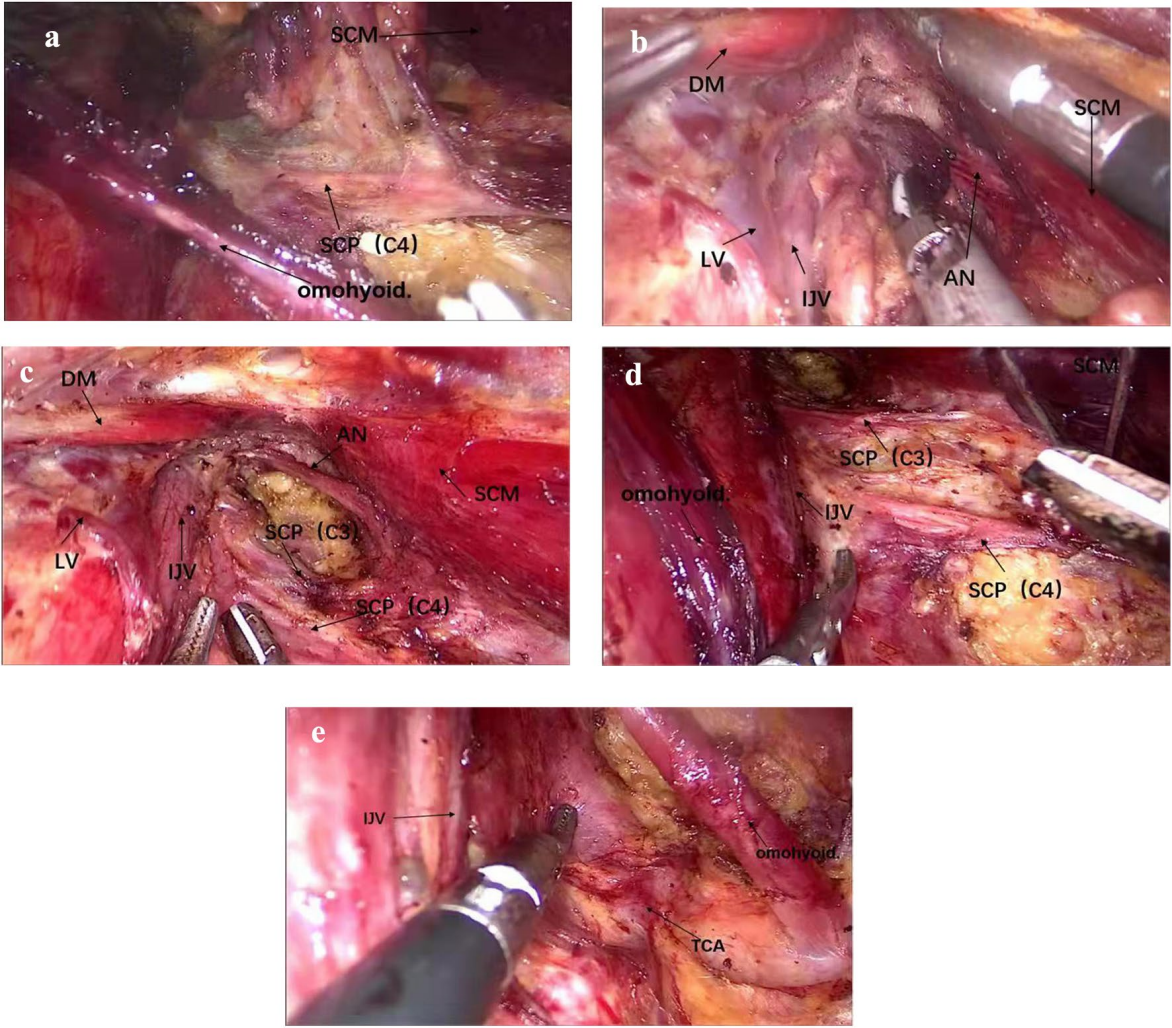
Step 6: the tissues of levels IV, III, and II are moved via breast approach. **a** The C4 nerve were protected carefully. **b** Dissect the carotid triangle. **c** Level II and accessory nerve after dissection. **d** Level III and C3, C4. **e** Level IV and the transverse carotid artery. *SCM* sternocleidomastoid muscle, *SCP* superficial cervical plexus, *DM* digastric muscle, *LV* lingual vein, *IJV* internal jugular vein, *AN* accessory nerve, *TCA* transverse carotid artery

Because the internal and inferior boundaries of the lateral compartment had been dissected (Fig. 6), all of the tissues from the lateral compartment could be easily dissected from level IV to level III to level II step by step. During these procedures, the C2-4 and accessory nerve were protected carefully.

7 The working space was washed, and drainage tubes were placed.                                                                                                                                                          

**Fig. 7:**
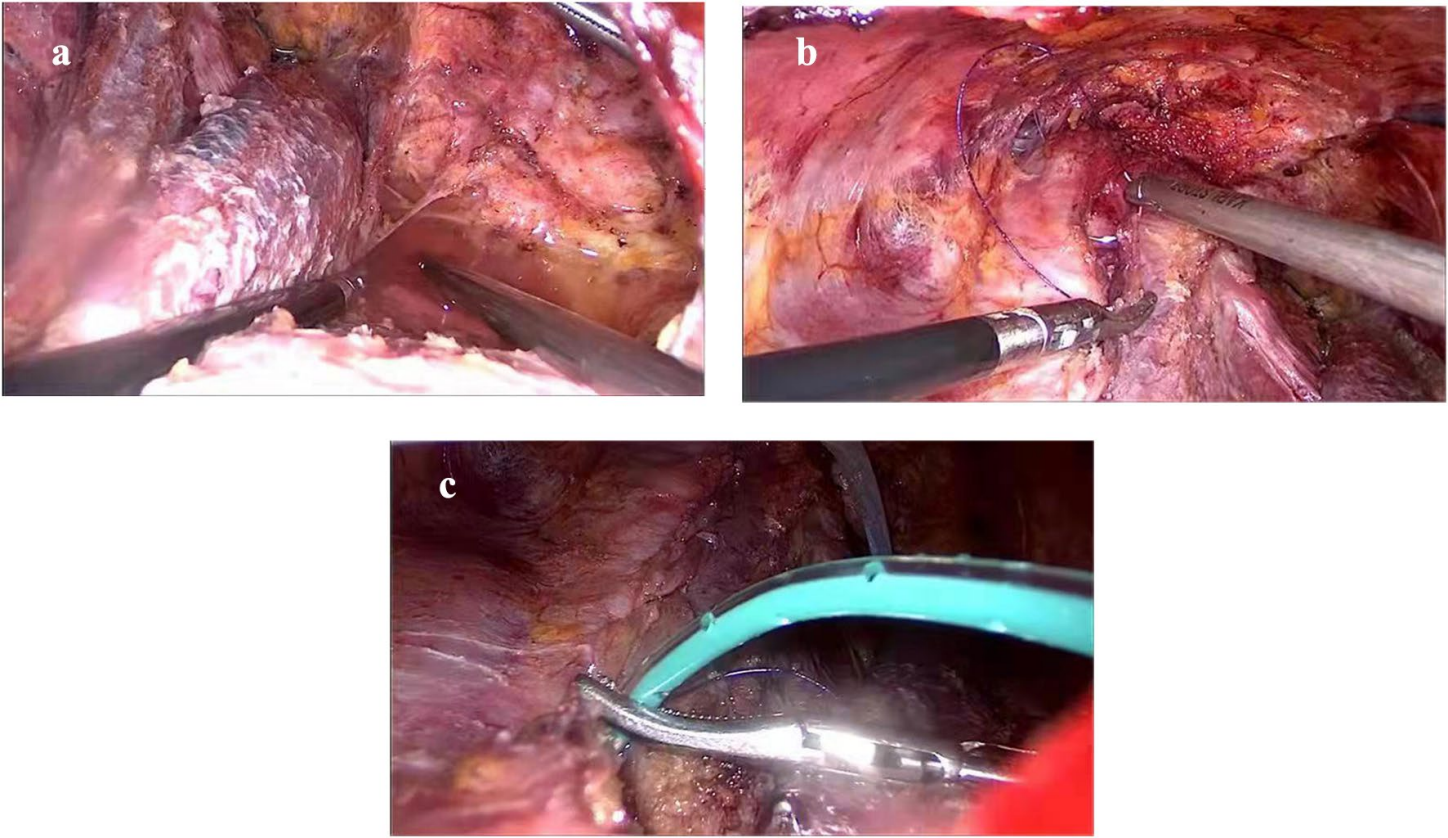
Step 7: working space is washed, and drainage tubes are placed. **a** The working space was washed with distilled water. **b** The cervical linea alba was sutured. **c** Drainage tubes were placed

The working space was washed with about 1000 mL of distilled water (Fig. 7). The cervical linea alba was sutured, and two drainage tubes were placed, one in the lateral compartment and one in the central compartment.

The operative procedure of the contrast procedure was not fixed. It was the same as Wu’s seven steps except in the following ways: (1) Thyroidectomy was performed before the isolation of the SCM and IJV. (2) Central lymph nodes were dissected via breast approach first and then dissected via oral approach supplementarily. (3) The IJV was carefully dissected from the cricoid cartilage down to the venous angle along the external border of the sternohyoid muscle. (4) In six cases in the control group, a 5 mm trocar in the middle and a 5 mm 30° laparoscope were used in the oral approach. Therefore, we needed to change from the 10 mm laparoscope to the 5 mm laparoscope at the beginning of the oral approach, and then change back to the 10 mm laparoscope upon the conclusion of the oral approach.

### Statistics

Quantitative variables were presented as the mean and standard deviation (Mean ± SD) whenever data proved to be normally distributed; otherwise, the median and interquartile range were used (interquartile range is provided in parentheses within the manuscript). The data were analyzed using t-tests in SPSS software (version 22.0). A P value of < 0.05 was considered to indicate statistical significance.

## Results

**Fig. 8:**
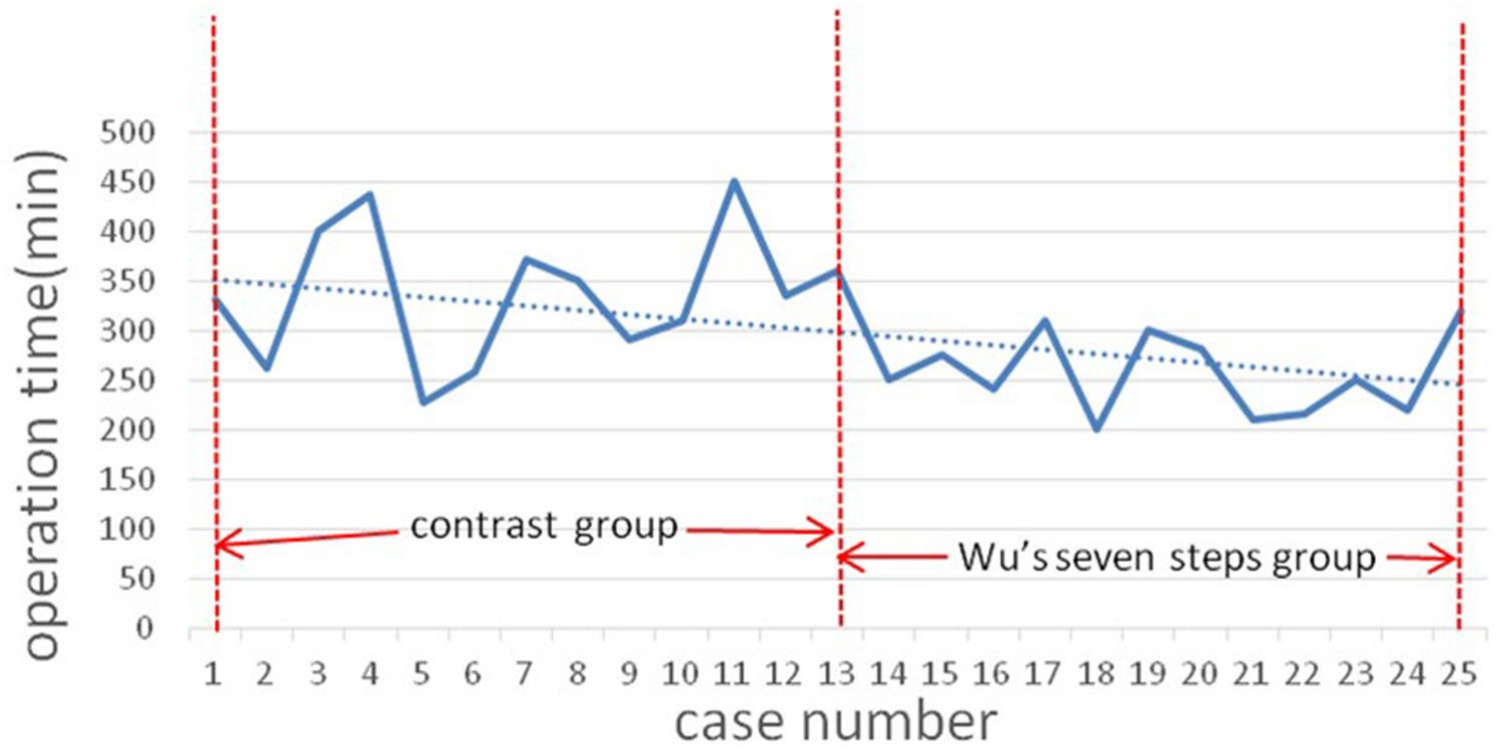
Operation time (min) of  the contrast group (from the case 1 to 13) and the Wu’s seven steps group (from the case 14 to 25)

The clinicopathological characteristics of the two groups are summarized in Table 1. Wu’s seven steps group included 12 patients (three men and eight women) with a mean age of 33.8 ± 9.1 years (range: 22–55 years). The control group included 13 patients (four men and nine women) with a mean age of 32.6 ± 11.1 years (range: 17–53 years). There was no statistical difference between the mean age of these two groups (*p* = 0.768). The two groups were similar in terms of gender ratio, mean tumor size, LND side, and duration of postoperative hospital stay. Although the occurrence of IJV injury in the control group was higher than in the Wu’s seven steps group (*p* = 0.165), there was no significant difference between the two groups. The operation time of the Wu’s seven steps group was 256.0 ± 41.0 min, shorter than that of the contrast group, which was 336.9 ± 68.0 min (*p* = 0.002, Fig. 8). The mean retrieved numbers of central and lateral lymph nodes were similar between the two groups (*p* = 0.243 and *p* = 0.198, respectively).

**Table 1:** Clinicopathological characteristics and operation times in the two study groups

	Wu’s seven steps group (*N* = 12)	Contrast group (*N* = 13)	*p* value
Age (years)	33.8 ± 9.1	32.6 ± 11.1	0.768
Age range (years)	22–55	17–53	
Male:Female	3:9	4:9	0.760
LND side (left:right)	4:8	9:4	0.078
TNM stage			0.480
I	11	13	
II	1	0	
Tumor size (mm)	19.1 ± 7.8	16.3 ± 9.1	0.419
Operation time (min)	256.0 ± 41.0	336.9 ± 68.0	0.002
Injury of IJV (case)	0	2	0.165
Postoperative hospital stay (days)	4.9 ± 0.8	5.0 ± 0.7	0.784
Number of retrieved LN			
Total central lymph nodes	11.3 ± 7.7	8.3 ± 4.7	0.243
Positive central lymph nodes	3.0 ± 2.6	5.0 ± 3.8	0.128
Total lateral lymph nodes	32.3 ± 10.3	27.1 ± 9.5	0.198
Positive lateral lymph nodes	7.0 ± 3.3	5.0 ± 2.9	0.636

The complication rates were similar between the two groups (Table 2). There were two cases of transient hypocalcemia in the Wu’s seven steps group and three cases in the contrast group (*p* = 0.704). In terms of transient hoarseness, there was one case in each group (*p* = 0.955). There were no cases of permanent hypocalcemia, recurrent laryngeal nerve injury, seroma formation, hemotoma formation, chyle leak, or wound infection in either group. Accessory nerve injury was found in one case in the Wu’s seven steps group (*p* = 0.308). Horner's syndrome was observed in one case of the contrast group postoperatively (*p* = 0.347). None of the differences of complications between these two groups were statistically significant.

**Table 2:** Comparison of complications in the two study groups

	Wu’s seven steps group	Contrast group	*p* value
Transient hypocalcemia	2	3	0.704
Permanent hypocalcemia	0	0	
Transient hoarseness	1	1	0.955
Permanent hoarseness	0	0	
Accessory nerve injury	1	0	0.308
Seroma formation	0	0	
Hematoma formation	0	0	
Chyle leak	0	0	
Wound infection	0	0	
Horner’s syndrome	0	1	0.347

## Discussion

In the wake of the development of endoscopic thyroidectomy and central compartment dissection in papillary thyroid cancer patients [10–14], more and more surgeons have begun attempting endoscopic lateral neck dissection [6, 15–17]. Most endoscopic lateral neck dissection were done by breast approach. Few surgeons did it by transoral approach [18]. Some modifications have been made to this technique as well [5]. Due to the connection between the manubrium of the sternum and the clavicle, it has been questioned whether metastatic lymph nodes can be dissected completely when they are located in the anterosuperior mediastinum (level VII) or corner region (level IV, where the IJV joins the subclavian vein) via breast approach [19, 20]. Previously, we tried endoscopic surgery via breast combined with oral approach to dissect the central and lateral compartments while avoiding the blockage of the sternal manubrium and clavicle, aiming to completely dissect the lymph nodes located in level VII and the inferior area of level IV [7]. However, the operation time was too long and the procedure was not easy to master, reflecting the need for a new strategy for this technique. Especially in male patients, it is difficult to operate because of the strong muscle of sternocleidomastoid muscle and the disturb from the apophysis of thyroid cartilage when the central compartment was dissected via transoral approach. In this study, the whole surgical procedure of endoscopic central and lateral neck dissection via breast combined with oral approach for papillary thyroid cancer was optimized and divided into seven steps, which are referred to as Wu’s seven steps.

In this study, the average operation time of the Wu’s seven steps group was 1 h less than that of the contrast group. We concluded that Wu’s seven steps can save some time by changing the direction of dissection on the surface of the IJV from down to up. This is because sometimes it takes more time to dissect the tissue from the carotid triangle first. If the lower part of the IJV is dissected, the dissection of the carotid triangle will be easier, and less time is required. Additionally, if the dissection of the central compartment is changed from the breast approach to the oral approach, the operation time can be reduced by some time or so. Furthermore, because Wu’s seven steps standardize the procedure, the operation can be proceeded more smoothly, and the operation time can be reduced as well. Wu’s seven steps also facilitate learning and communication between surgeons.

IJV injury could easily occur in endoscopic lateral neck dissection [6, 21]. If IJV bleeding occurs, small lacerations can be sutured with a 5-0 or 6-0 proline wire, or hemlock clips can be used for vascular branches. If bleeding cannot be controlled, a transverse incision may help repair larger lacerations under direct vision. The incidence rate of IJV injury in Wu’s seven steps was lower than in the contrast group, although the difference was not statistically significant. In the Wu’s seven steps group, the IJV was dissected to expose the anterior and external boundaries from the venous angle up to the cricoid cartilage along the external border of the sternohyoid muscle, but in the contrast group, the IJV was dissected from up to down, and the lymph nodes at the carotid triangle were dissected first. The demerit of this contrast approach is that it misleads the dissection to the back of IJV easily, thus increasing the risk of IJV injury. However, when the IJV is dissected from down to up, the dissection would performed in front of the IJV, so the injury of IJV is less likely to happen.

Accessory nerve injury was found in the Wu’s seven steps group as a result of heat injury from the harmonic scalpel. Horner’s syndrome was observed in one case of the contrast group postoperatively, and it was suspected that the cervical sympathetic nerve may have been injured by the heat from the harmonic scalpel when the lymph node near the carotid sheath was dissected. None of the differences in complications between these two groups were statistically significant.

According to the other surgical complications of these two groups, we found that the recurrent laryngeal nerve injury rate and hypoparathyroid function incidence were statistically similar in these two groups. There was no hematoma formation, chyle leak, postoperative infection, or phrenic nerve injury in either group. The reason for there being no statistically significant differences in surgical complications may be the small number of study cases. The chin paresthesia has been a common symptom in both Wu’s seven steps and contrast group, it persist for a period less than 6 months because of the transoral approach. The time of chin paresthesia is shorter in this combined approach than in the transoral vestibular approach (TOETVA), because the oral insicion is shorter and the tunnel of the laporascopic is narrower in combined approach than in TOETVA.

## Conclusion

In conclusion, this study revealed that the procedure comprising Wu’s seven steps for endoscopic central and lateral neck dissection via breast combined with oral approach for papillary thyroid cancer treatment is a feasible and safe approach and achieves excellent cosmetic results. The advantages of Wu’s seven steps are the good oncological effect and optimized procedure. This approach could be an ideal choice for patients with lateral lymph node metastasis who are young or those who strongly desire a ‘scarless approach.’
